# Molecular and Genomic Characterization of *Vibrio mimicus* Isolated from a Frozen Shrimp Processing Facility in Mexico

**DOI:** 10.1371/journal.pone.0144885

**Published:** 2016-01-05

**Authors:** Iliana Guardiola-Avila, Evelia Acedo-Felix, Itzel Sifuentes-Romero, Gloria Yepiz-Plascencia, Bruno Gomez-Gil, Lorena Noriega-Orozco

**Affiliations:** 1 Centro de Investigación en Alimentación y Desarrollo, A.C Hermosillo, Sonora, México; 2 Mazatlán Unit for Aquaculture and Environmental Management. Centro de Investigación en Alimentación y Desarrollo, A.C. Mazatlán, Sinaloa, México; 3 Guaymas Unit: Quality Assurance and Management of Natural Resources. Centro de Investigación en Alimentación y Desarrollo, A.C. Guaymas, Sonora, México; University of North Carolina at Charlotte, UNITED STATES

## Abstract

*Vibrio mimicus* is a gram-negative bacterium responsible for diseases in humans. Three strains of *V*. *mimicus* identified as *V*. *mimicus* 87, *V*. *mimicus* 92 and *V*. *mimicus* 93 were isolated from a shrimp processing facility in Guaymas, Sonora, Mexico. The strains were analyzed using several molecular techniques and according to the cluster analysis they were different, their similarities ranged between 51.3% and 71.6%. ERIC-PCR and RAPD (*vmh*390R) were the most discriminatory molecular techniques for the differentiation of these strains. The complete genomes of two strains (*V*. *mimicus* 87, renamed as CAIM 1882, and *V*. *mimicus* 92, renamed as CAIM 1883) were sequenced. The sizes of the genomes were 3.9 Mb in both strains, with 2.8 Mb in ChI and 1.1 Mb in ChII. A 12.7% difference was found in the proteome content (BLAST matrix). Several virulence genes were detected (e.g. capsular polysaccharide, an accessory colonization factor and genes involved in quorum-sensing) which were classified in 16 categories. Variations in the gene content between these genomes were observed, mainly in proteins and virulence genes (e.g., hemagglutinin, mobile elements and membrane proteins). According to these results, both strains were different, even when they came from the same source, giving an insight of the diversity of V. mimicus. The identification of various virulence genes, including a not previously reported *V*. *mimicus* gene (*acf*D) in ChI in all sequenced strains, supports the pathogenic potential of this species. Further analysis will help to fully understand their potential virulence, environmental impact and evolution.

## Introduction

Generally, *Vibrio* species are accountable for the diseases associated to the natural bacterial flora of aquatic environments or seafood [1]. Several species of this genus can produce illness in humans mainly *Vibrio cholerae*, *Vibrio parahaemolyticus* and *Vibrio vulnificus*. *Vibrio mimicus* has been associated with human diseases such as gastroenteritis, ear infections and severe cholera-like diarrhea; with symptoms such as diarrhea, nauseas, vomiting, abdominal pain and sometimes fever [2–9]. This bacterium has been isolated from a large variety of seafood, such as oysters, turtle eggs, shrimp, crabs, snails, lobsters, and fish, as well in water samples, sediment and plants [2,5,6,10–13]. Similar to other *Vibrio* species, *V*. *mimicus* has two circular chromosomes of different size, a larger (ca. 3.0 Mb) chromosome and a smaller (ca. 1.5 Mb) chromosome [14–16]. The genes required for growth and viability are in the larger chromosome, while the genes for adaptation to environmental change are in the smaller [16]. Also, multiple virulence factors had been detected in both chromosomes, combinations of which regulated the pathogenic potential [3,16–17]. The genetic diversity of *Vibrio* species, including *V*. *mimicus*, isolated form different sources has been documented [16,18,19]. It has also been reported that *V*. *mimicus* could represent the genetic reservoir of virulence genes for other *Vibrio* species or to be recipients of gene transfers [19–21]. Thus, the identification of their virulence factors could provide information of the biology and evolution of an organism [22], and also of genetic diversity in the same environmental niche.

Numerous molecular techniques such as random amplified polymorphic DNA (RAPD), repetitive extragenic palindromes (REP), and amplified ribosomal DNA restriction analysis (ARDRA), along with real-time PCR, DNA sequencing, and microarrays have been applied to detect, identify and characterizer microorganisms for epidemiological purposes and allow to study and understand their pathogenicity and virulence [23,24]. DNA sequencing technologies had become more available, thus the generation of genomic information is growing, therefore it is important to compare the new genomes with the existing sequences, comparative genomic, to identify both novel and conserved features, and be able to determine genotype-phenotype relationships and explore what organisms really do with their genetic potential [22,25].

A molecular characterization of three strains of *V*. *mimicus* isolated from a processing facility for frozen shrimp was conducted to analyze their similarity and/or differences. Additionally, the complete genome sequence of the two strains that showed less similarity was obtained to estimate their pathogenic potential, were bioinformatics analyses were performed. These molecular and genomic characterizations could give us insight of the diversity of this species.

## Materials and Methods

### Bacteria strains

Three strains of *V*. *mimicus* were isolated from the water at the washing step in a freezing-shrimp company in Guaymas, Sonora, Mexico during September 2012. Water samples were collected with the permission of the processing company owner. The strains were isolated in thiosulfate citrate bile salts sucrose (TCBS) agar, and biochemical characterization was performed before PCR identification. The strains were designated as *V*. *mimicus* 87 (Vm 87), *V*. *mimicus* 92 (Vm 92) and *V*. *mimicus* 93 (Vm 93). *V*. *mimicus* type strain CAIM 602^T^ (ATCC 33653) was used as positive control for all the tests of this study.

### DNA extraction and bacterial identification

DNA was obtained from a fresh pure culture in trypticase soy broth (TSB, 34°C/18 h), as described previously with some modifications [26]. The procedure basically consisted of enzymatic lysis of the cells, extraction with phenol/chloroform/isoamyl alcohol, ethanol precipitation, and resuspension of DNA in TE buffer (10 mM Tris-HCl, 1 mM EDTA, pH 8.0) with RNAse (20 mg/ml). The DNA extraction was confirmed by agarose gel electrophoresis (0.8%), and DNA was stored at -20°C for further use.

*V*. *mimicus* isolates were confirmed by the amplification of the hemolysin (*vmh*) gen [27,28] using the following primers: vmh390F (GGTAGCCATCAGTCTTATCACG) and vmh390R (ATCGTGTCCCAATACTTCACCG). PCR amplification was performed in a thermal cycler (Perkin Elmer 480) with the following temperature profile: an initial denaturation at 95°C for 5 min; 35 cycles of 30 s at 95°C, 30 s at 53°C and 30 s at 72°C; and a final extension at 72°C for 5 min. Amplified DNA was visualized using agarose gel electrophoresis (1.8%).

### Molecular analysis

PCR confirmed *V*. *mimicus* isolates were analyzed using the following molecular methodologies.

#### Amplified ribosomal DNA restriction analysis (ARDRA)

PCR amplification of the 16S rRNA using the universal primers 27-F (AGAGTTTGATCCTGGCTCAG) and 1492-R (GGTTACCTTGTTACGACTT) was performed. PCR amplification was done in a thermal cycler (Perkin Elmer 480) with the following temperature profile: an initial denaturation at 96°C for 5 min; 36 cycles of 1 min at 94°C, 1 min at 50°C and 1 min at 72°C; and a final extension at 72°C for 5 min. PCR products were visualized in 1.2% agarose electrophoresis gel. Afterwards, the amplified products were treated with the following restrictions enzymes: *Cfo*I, *Hae*III, *Hinf*I, *Hap*II and *Alu*I. Ten microliters of PCR product were digested with restriction enzymes (1 μL of restriction enzyme, 2 μL of specific buffer 10X, 1 μL of albumin and 9 μL of sterile water) and incubated at 37°C for 18 hr according to the manufacturer´s instructions. The digested products were visualized by electrophoresis in an agarose gel (1.8%) at 95 V for 90 min.

#### Repetitive Element PCR (Rep-PCR)

Two different techniques were utilized: Enterobacterial repetitive intergenic consensus PCR (ERIC-PCR) [29] and (GTG)_5_-PCR [30]. The primers used for the Rep-PCR were ERIC-1 (5′-ATGTAAGCTCCTGGGGATTCAC-3′) and ERIC-2 (5′-AAGTAAGTGACTGGGGTGAGCG-3′) for ERIC-PCR and (GTGGTGGTGGTGGTG) for (GTG)_5_-PCR. PCR amplification was performed in a thermal cycler (Perkin Elmer 480) with the following temperature profile: initial denaturation at 94°C for 6 min; 35 cycles of 30 s at 94°C, 60 s at 48°C and 5 min at 72°C; and a final extension at 72°C for 7 min. PCR products were separated by electrophoresis in an agarose gel (1.2%) at 90 V for 100 min.

#### Random Amplified Polymorphic DNA PCR (RAPD)

Two different primers were used: OPI-3 (5’CAGAAGCCCA3’) and *vmh*390R (ATCGTGTCCCAATACTTCACCG). The PCR amplifications were performed in a thermal cycler (Perkin Elmer 480), with the OPI-3 primer the following temperature profile was used: an initial denaturation at 94°C for 4 min; 30 cycles of 1 min at 94°C, 1 min at 37°C and 1 min at 72°C; and a final extension at 72°C for 6 min. For the *vmh*390R primer, the same program described by Bi *et al*. (2000) was used. PCR products were visualized by electrophoresis in an agarose gel (1.8%) at 95 V for 90min.

#### Fingerprint analysis

Gel digital images were captured with Image Lab software (BIO-RAD, Molecular imager: Gel Doc^TM^ XR+) and were analyzed with BioNumerics software (Applied Maths, Inc.). A cluster analysis was performed by calculating a similarity/distance matrix with DICE, a similarity coefficient based on band presence or absence. Then, the resulting similarity matrix was converted into a dendrogram with a clustering algorithm by Unweighted Pair Group Method with the Arithmetic Mean (UPGMA) method with optimization set at 1%.

### Genome Sequence

Based on the molecular characterization, two different *V*. *mimicus* strains were sequenced using a semiconductor NGS platform (Ion Torrent Personal Genome Machine sequencer, Life Technologies) with a 316 chip at CIAD Mazatlán. The reads were assembled with the program Newbler ver.2.3 mapping against CAIM 602^T^ [31]. A genome-wide assembly and contig synteny was constructed with Mauve Genome Alignment software ver. 2.3.1 [32] using *V*. *mimicus* 451 as a reference strain[16]. The contigs were further reassembled with Geneious R6 ver. 6.0.3 (Biomatters Ltd) to obtain two chromosomes (ChI and ChII). Original contigs were annotated by RAST [33] (http://rast.nmpdr.org/) and by NCBI (http://www.ncbi.nlm.nih.gov). We constructed a data base with the information from the annotation for the study of the differences and similarities.

#### Phylogenetic reconstruction

Phylogenetic reconstructions of 28 core genome virulence genes of both chromosomes of *V*. *mimicus* CAIM 1882, 1883, 602 and *V*. *mimicus* 451 were done using two methods: Maximum likelihood (ML) and Neighbor joining (NJ). The ML and NL phylogenetic trees were obtained using MEGA (ver. 5.1) and the robustness of each topology was checked by 1000 bootstrap replicates. *V*. *cholerae* O1 biovar El Tor N16961 [34] was used as outgroup in both methodologies. Seventeen sequences of virulence genes were selected from ChI: an accessory colonization factor (AcfD), capsular polysaccharide synthesis enzyme (CpsABCD), chitinase, two hemolysins, MSHA biogenesis protein (MshEFGIJKLMN), MSHA pilin protein (MshABCD), outer membrane proteins (OmpK, OmpT, OmpU), polysaccharide export lipoprotein (Wza), protease IV, regulatory protein (LuxO), transcriptional activator (ToxR), transcriptional regulator (LuxR), transmembrane regulatory protein (ToxS) and type IV pilus (PilMNOPQ). While in ChII eleven sequences of virulence genes were chosen: an autoinducer 2 (LuxQ, LuxP), chitin binding protein, chitinase, hemolysin (HlyA), hemolysin, outer membrane protein (OmpW), putative hemolysin, sensor histidine kinase (CqsS), thermolabile hemolysin and transcriptional regulator (LuxR).

### Comparative Microbial Genomics (CMG)

The genomes of *V*. *mimicus* were analyzed by the CMG-Biotools [35] to obtain a BLAST matrix. The BLAST hit was considered significant if 50% of the alignment consists of identical matches and the length of the alignment is 50% of the longest gene.

## Results

**Fig 1:**
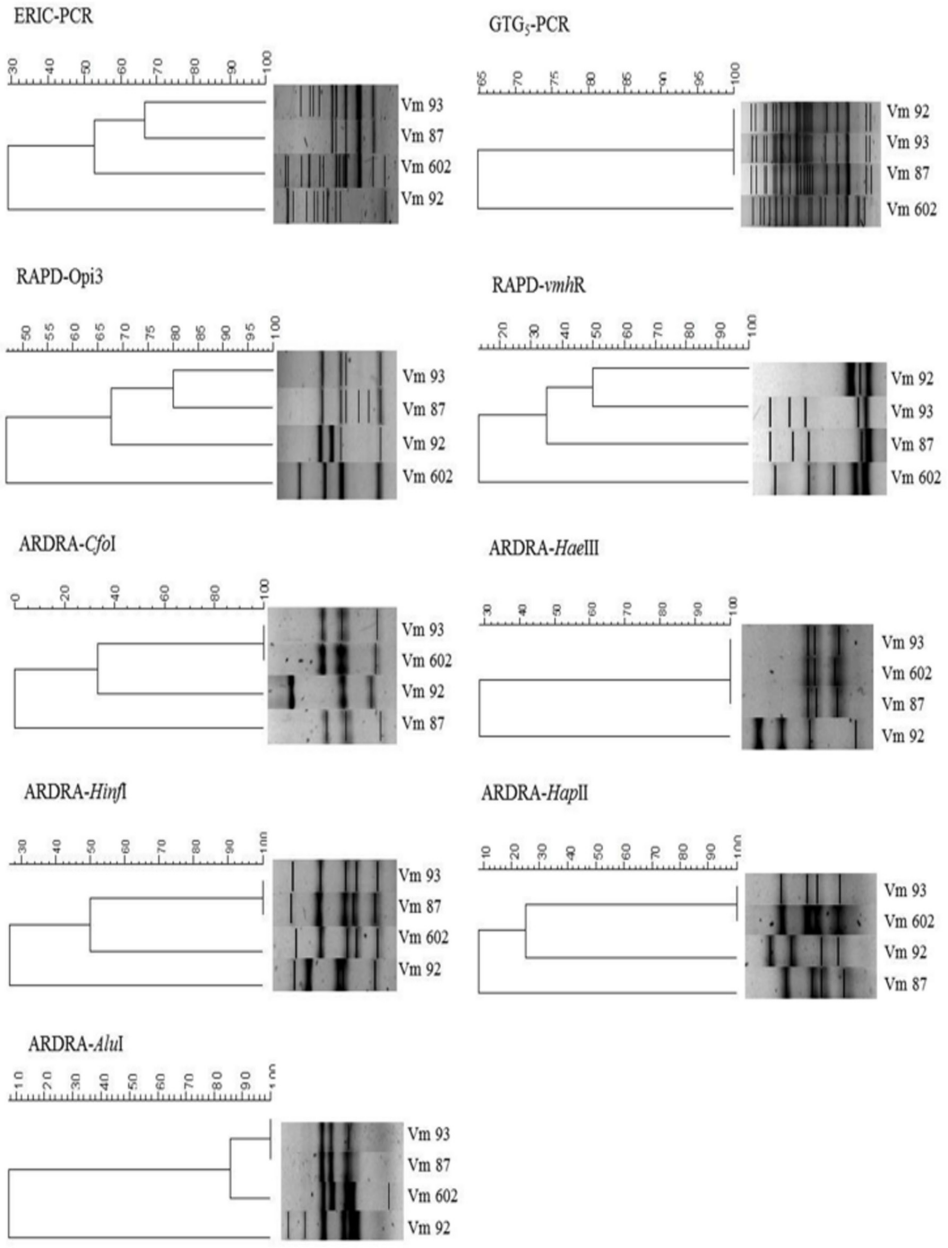
Dendrograms of ERIC-PCR, RAPD, GTG-5PCR and ARDRA used for the analysis of *V*. *mimicus* 87, 92, 93 and CAIM 602 (type strain). The images were analyzed with Bionumerics software (Applied Maths, Inc.) with Dice correlation coefficient for the distance matrix and UPGMA with optimization set at 1% to create the dendrogram.

All three isolates showed the 390 bp fragment corresponding to the *vmh* gene, and were thus confirmed as *V*. *mimicus*. The gels obtained from the different molecular fingerprinting methodologies (ERIC-PCR, RAPD, GTG-_5_ and ARDRA) showed different patterns between isolates and the type strain (CAIM 602^T^). The dendrograms from the cluster analysis of the gels from each methodology were obtained (Fig 1).

**Fig 2:**
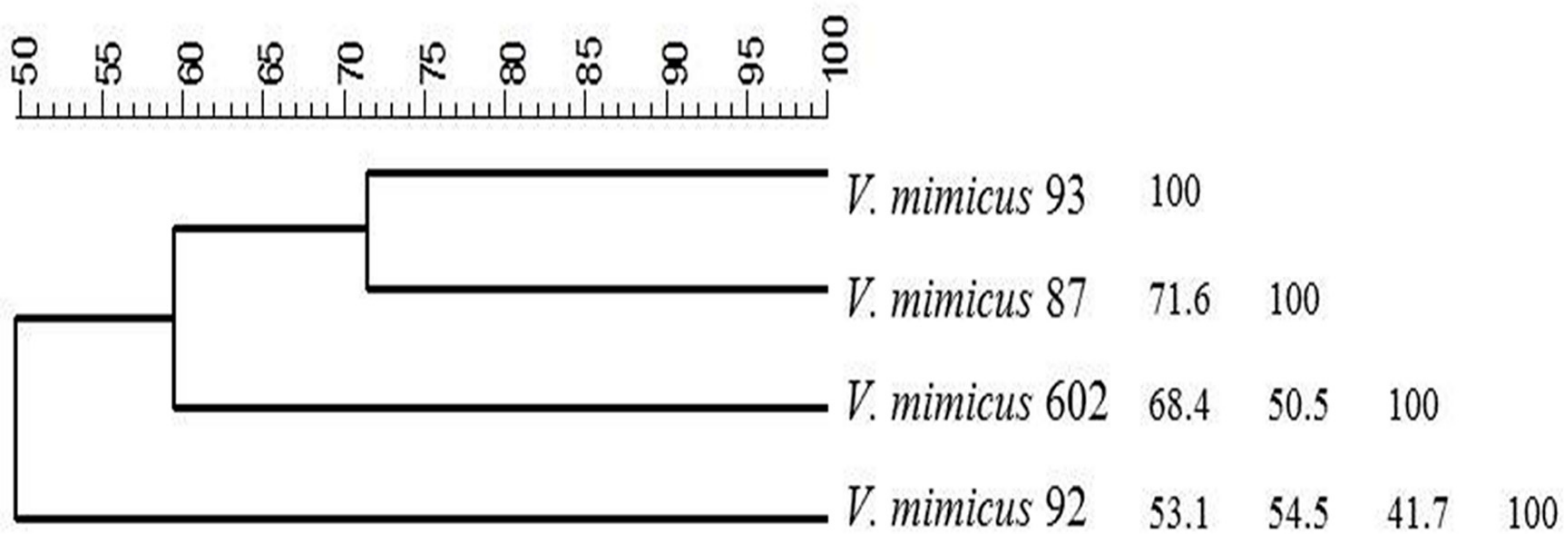
Composite dendrogram for the three strains of *V*. *mimicus* isolated from frozen shrimp process (*V*. *mimicus* 87, 92 and 93) and *V*. *mimicus* CAIM 602T (type strain). The dendrogram and a similarity matrix were obtained by a cluster analysis of ERIC-PCR, GTG-5, RAPD and ARDRA (Bionumerics, Applied Maths, Inc).

With ERIC-PCR, the strains yielded 5 to 13 amplified products of different sizes ranging from approximately 0.1 kb to 4.0 kb, and the similarities ranged between 30% and 66% among the isolates. With RAPD-*vmh*390R the strains produced 3 to 5 bands ranging in size from approximately 0.3 kb to 2.0 kb, and the similarities ranged between 36% and 50%, and with RAPD-Opi3 the strains yielded 4 to 6 amplified products of different sizes ranging from approximately 0.2 kb to 3.0 kb, and the similarities ranged between 67% and 80% among the isolates. With ARDRA, the analysis results showed that *Cfo*-I and *Hap*-II were the most discriminatory enzymes; with both enzymes, the strains generated 3 to 4 amplified products with a size ranging from approximately 0.2 kb to 0.9 kb for *Cfo*-I, while for *Hap*-II sizes ranged from 0.1 kb to 0.6 kb; and the similarity among isolates were ranged between 0% and 35% for *Cfo*-I and 10% and 26% for *Hap*-II. With GTG_5_-PCR no differences among these isolates were found. In addition, a composite dendrogram and a similarity matrix was obtained by a cluster analysis of all the band patterns obtained from the different methodologies used (Fig 2). From these data, Vm 92 was the most different from the others, with Vm 87 presented only 54.5% similarity, and with Vm 93 showed 41.7% similarity; whereas Vm 87 and Vm 93 had 71.6% similarity.

From this molecular characterization, it was confirmed that these three isolates were indeed different strains of *V*. *mimicus*. Two of them (i.e., Vm 87 and Vm 92) were selected for sequencing so it can be estimate their pathogenic potential. These strains were deposited at the Collection of Aquatic Important Microorganisms (CAIM: www.ciad.mx/caim) and were registered as CAIM 1882 (Vm 87) and CAIM 1883 (Vm 92). Both genomes were deposited in GenBank under accession numbers PRJNA219179 and PRJNA219181, respectively.

The sequencing yielded a total of 2,932,276 reads (mean length 183 bp) for a total of 540 Mb and an average coverage of 125.6X for CAIM 1882. For CAIM 1883, a total of 2,773,308 reads were obtained (mean length 193 bp) for a total of 536 Mb and an average coverage of 124.5X. The reads were assembled, and 434 contigs for CAIM 1882 (N50 40,957 bp, 92.58% reads mapped) and 455 contigs for CAIM 1883 (N50 41,061 bp, 92.76% reads mapped) were obtained. A summary of the general features from the assembled genomes by chromosome of CAIM 1882 and CAIM 1883 are shown in Table 1.

**Table 1:** Summary of the general features of *V*. *mimicus* CAIM 1882, CAIM 1883, CAIM 602^T^ and *V*. *mimicus* 451.

		CAIM 1882	CAIM 1883	CAIM 602^T^	*V*. *mimicus* 451
**Chromosome I**	size (Mb)	2,819,391	2,820,150	2,907,560	2,972,217
	rRNAs	7	7	7	7
	tRNAs	72	70	71	93
	CDS	2620	2606	2639	2670
	%GC	46.8	46.8	46.6	46.6
**Chromosome II**	size (Mb)	1,141,600	1,115,258	1,405,203	1,304,309
	rRNAs	0	0	0	0
	tRNAs	5	6	6	4
	CDS	1092	1019	1383	1234
	%GC	46.6	46.5	46.2	45.7
**Total Genome (Mb)**		3,960,991	3,935,408	4,312,763	4,276,526

Mb = mega base pairs; rRNA = ribosomal ribonucleic acid; tRNA = transfer ribonucleic acid; CDS = coding sequence; %GC = percentage of guanine-cytosine content.

**Fig 3:**
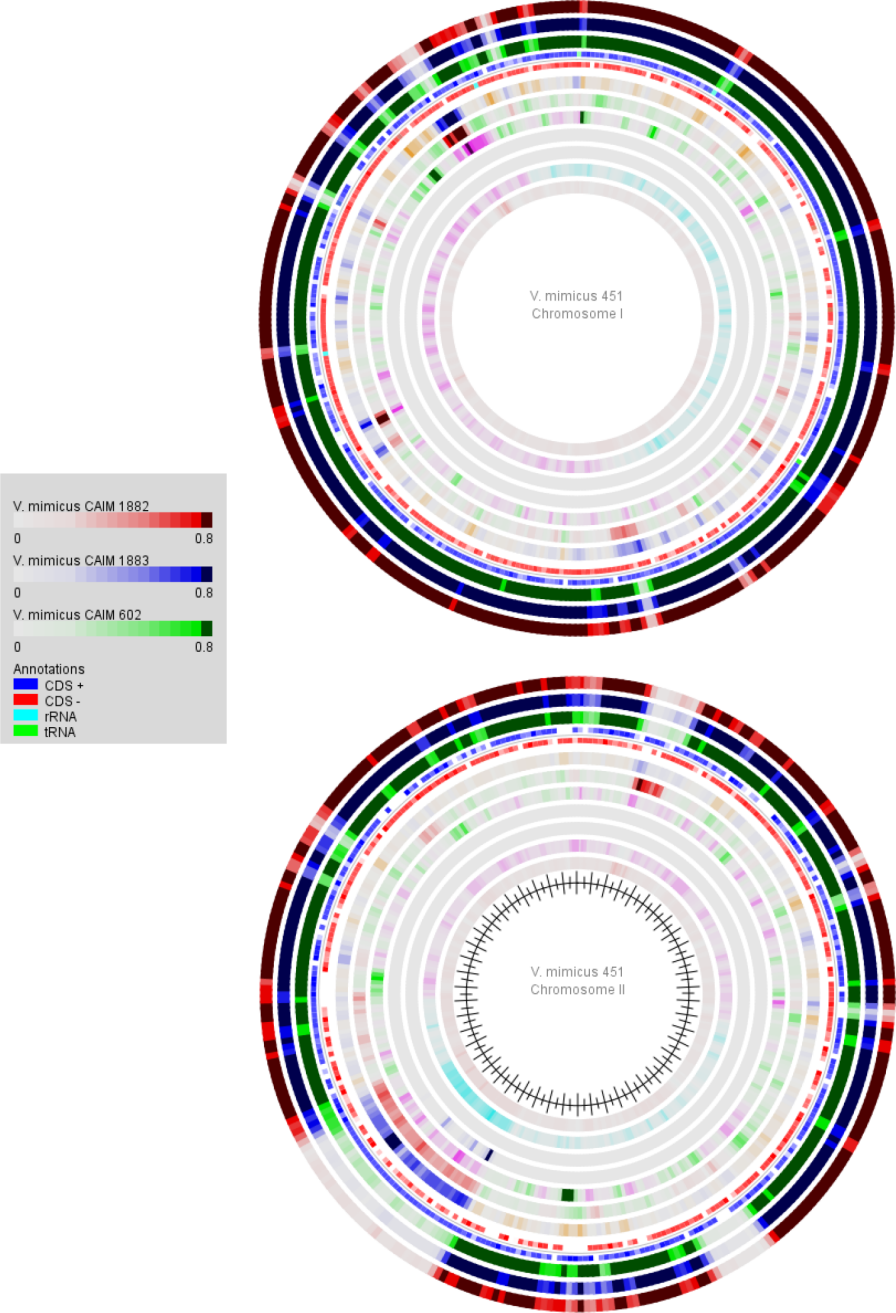
Genome Atlas obtained of *V*. *mimicus* CAIM 602 (green), CAIM 1882 (red), CAIM 1883 (blue) and *V*. *mimicus* 451 (as control strain). The atlas was constructed with GeneWiz Browser 0.94. From the inner ring to the outer ring: Percent AT, GC Skew, Global Inverted Repeats, Global Direct Repeats, Position Preference, Stacking Energy and Intrinsic Curvature.

A genome atlas was generated with GeneWiz browser, where the differences between the genomes of *V*. *mimicus* CAIM 602, CAIM 1882, CAIM 1883 and *V*. *mimicus* 451 can be visualized (Fig 3). A major variability was observed in ChII than in ChI, some of the differences found in ChI were in genes such as Type IV fimbrial assembly (PilB), zona occludens toxin, accessory cholera enterotoxin, type II restriction enzymes, phage integrase, polysaccharide biosynthesis proteins, transcriptional regulators, and hypothetical proteins. While in ChII genes such as membrane proteins, transcriptional regulators, flagellar proteins, integron integrase, transport proteins, mobile elements, multidrug resistance efflux pumps, and hypothetical proteins were found.

**Fig 4:**
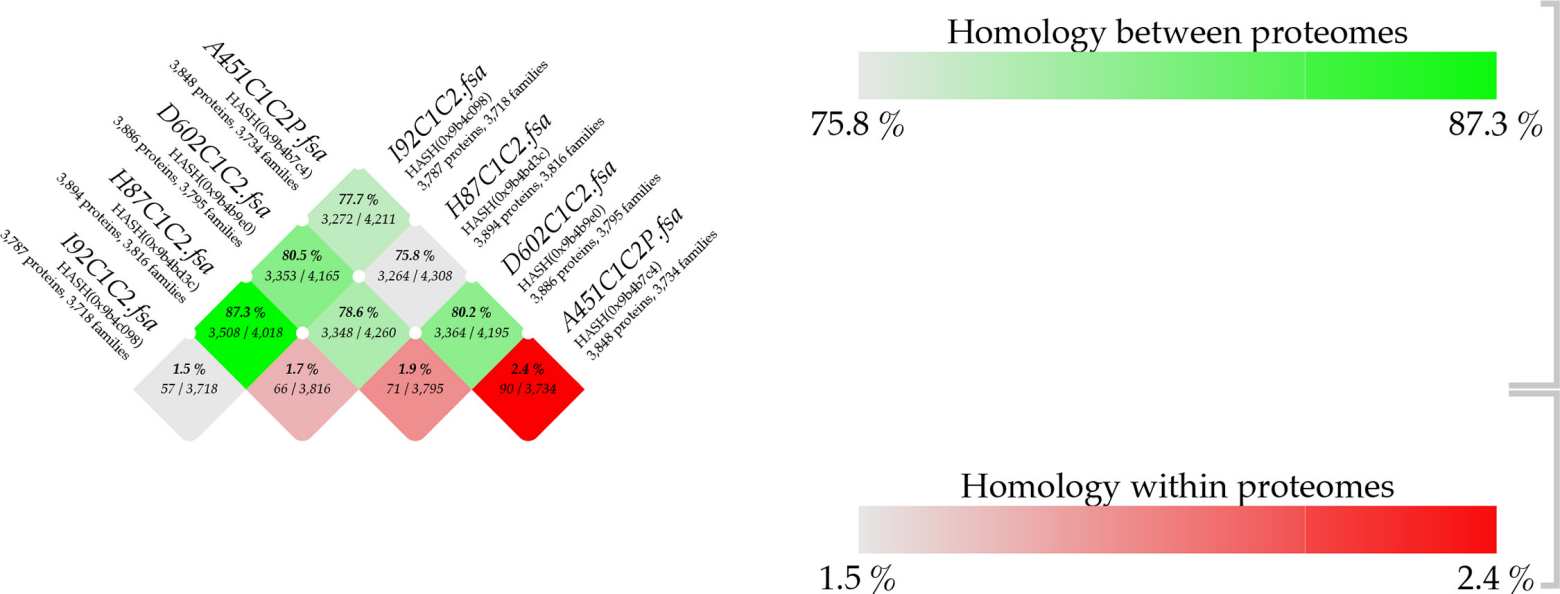
BLAST matrix (proteome comparison) of *V*. *mimicus* 451, *V*. *mimicus* CAIM 602, *V*. *mimicus* CAIM 1882 and *V*. *mimicus* CAIM 1883. The BLAST matrix was obtained using CMG-biotools [35].

A proteome comparison was obtained with the four genomes of *V*. *mimicus* (> 50% homology) and represented in a BLAST matrix (Fig 4). The homology between the proteomes ranged from 75.8% to 87.3%; observing differences among clinical and environmental strains, and even between environmental strains. Whereas, the homology within proteomes (number of proteins that have homologous hits within the proteome itself) was among 1.5% and 2.4%, being the environmental strains (CAIM 1182 and 1183) those with the lowest percentages.

A summary of the virulence genes found by category in each strain according to the classification used by Kimes *et al*. [36] is shown in Table 2. CAIM 1882 showed slightly more virulence genes than CAIM 1883. For chromosome I (ChI) a higher number of genes was present in the category type I secretion proteins, followed by flagellar proteins, extracellular components, potential regulators and type IV secretion proteins. While in chromosome II (ChII), the main categories were the type I secretion proteins, chemotaxis proteins, antibiotic resistance proteins and iron uptake.

**Table 2:** Summary of virulence genes detected in *V*. *mimicus* CAIM 1882 and CAIM 1883 by category.

	*V*. *mimicus* CAIM 1882		*V*. *mimicus* CAIM 1883	
Virulence Category	ChI	ChII	ChI	ChII
Chemotaxis Proteins	34	33	34	33
Flagellar Proteins	52	0	52	0
Antibiotic Resistance Proteins	29	19	29	20
Hemolysins	9	8	8	8
Toxins	1	3	2	4
Proteases	25	6	25	6
Iron uptake	21	23	21	20
Extracellular components	45	17	46	15
General Stress Response Proteins	25	18	24	17
Type 1 Secretion Proteins	140	57	140	56
Twin-arginine translocation pathway	3	0	3	0
Type 2 Secretion Proteins	24	8	24	8
Type IV Pilus Proteins	40	11	40	11
Type VI Secretion Proteins	5	8	6	8
Potential Regulators	41	10	41	10
Mobile elements	8	7	6	8
**Total**	**502**	**228**	**501**	**224**

ChI = chromosome I; ChII = Chromosome II

Moreover, examples of genes encoding virulence factors detected in ChI in both strains were hemolysins, proteases, outer membrane proteins [OmpU, OmpT, OmpK and OmpV], a type IV and MSHA pilus, an aerobactin siderophore, a capsular polysaccharide, an accessory colonization factor (*acf*D), the transmembrane regulatory protein ToxS, the transcriptional activator ToxR and presence of quorum-sensing regulation system (LuxS, LuxO, LuxR). In addition, some proteins and phage shock proteins were found. Whereas in ChII of both strains, examples of genes coding for virulence factors were metalloproteases, chemotaxis proteins, as well as various hemolysins (e.g., cytolysin and hemolysin HlyA, thermolabile hemolysin precursor and thermostable hemolysin delta-VPH), a putative phosphatase, an adhesin, a chitinase, a type II/IV/ and type VI secretion system proteins. Additionally, genes involved in quorum-sensing (autoinducer 2 (AI-2), CAI-1 autoinducer synthase, sensor histidine kinase CqsS), and various hypothetical proteins were found. In addition, both strains possessed an integron and two prophages in ChII, but no plasmids were detected in any of them.

**Fig 5:**
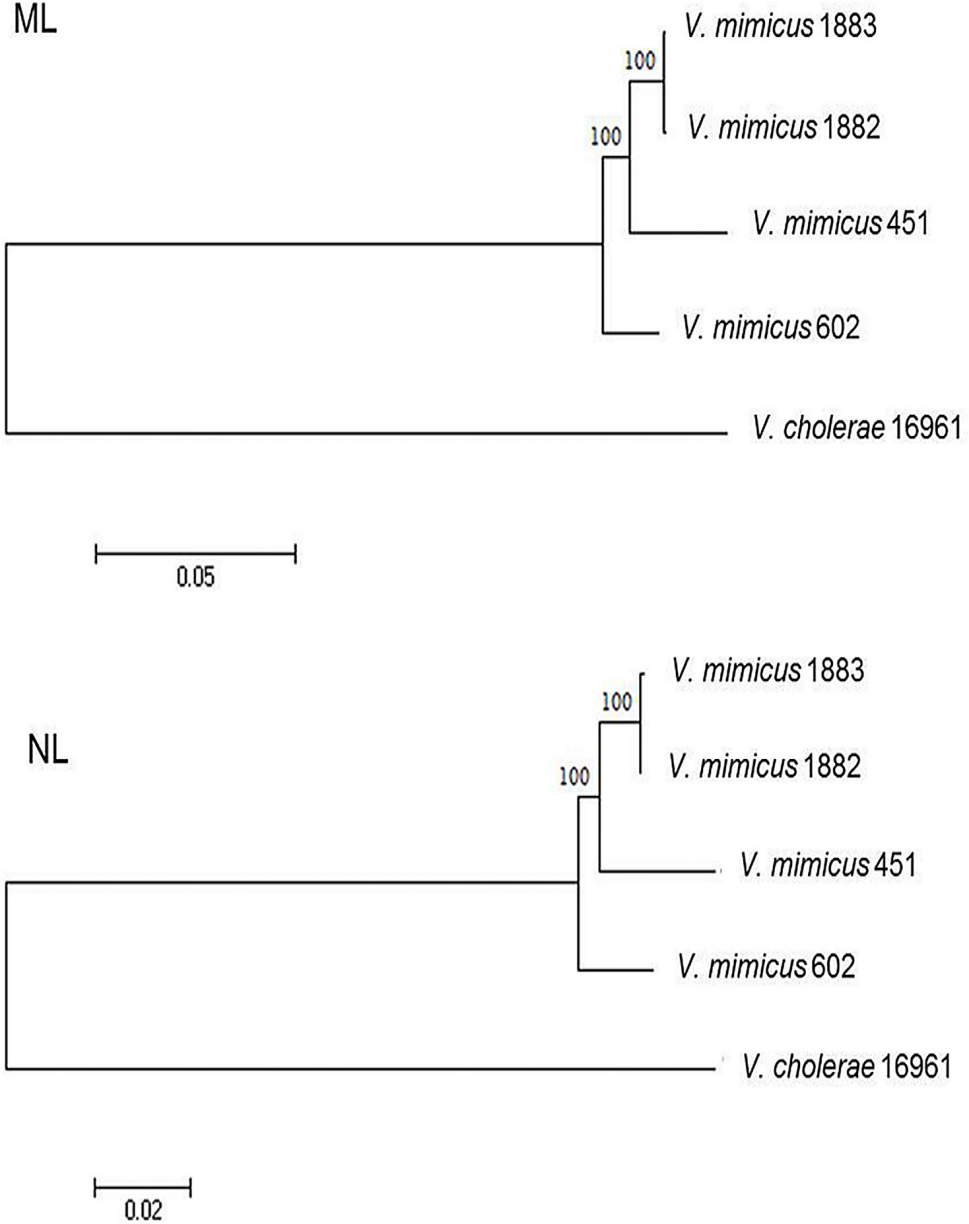
Maximum likelihood (ML) and Neighbor joining (NJ) phylogenetic trees of 28 core genome virulence genes. The ML and NL phylogenetic trees of both chromosomes of *V*. *mimicus* CAIM 1882, 1883, 602 and *V*. *mimicus* 451 were obtained using MEGA, where *V*. *cholerae* O1 biovar El Tor N16961 [34] was used as outgroup.

Based on the virulence genes detected for *V*. *mimicus* (CAIM 1882 and 1883), 28 core genome genes of clinical importance in both chromosomes were selected for a phylogenetic study (Fig 5). As expected, a similar cluster pattern for the ML and NJ trees was observed, and both environmental strains were grouped together.

Furthermore, the complete list of gene variations in each strain is shown in Table 3. In ChI few differences were found, which included differences in virulence genes and hypothetical proteins; while in ChII, more genes were detected in CAIM 1882 than in CAIM 1883, and the differences were mainly in virulence genes, transcriptional regulators and hypothetical proteins.

**Table 3:** Differences in gene content in *V*. *mimicus* CAIM 1882 and CAIM 1883.

	CAIM 1882		CAIM 1883	
**Chromosome I**	TPR domain protein in aerotolerance operon	1	Hcp protein	1
	Soluble lytic murein transglycosylase	1	WbfB protein	1
	Mll3428 protein	1	GGDEF family protein	1
	Streptococcal hemagglutinin protein	1	Putative transcriptional regulator LysR	1
	Alpha-1,2-mannosidase	1	VgrG protein	1
	Mobile element protein	2	Protein of unknown function DUF1254	1
	Hypothetical proteins	19	RTX toxin putative	1
			Methyl-accepting chemotaxis protein I	1
			Hypothetical proteins	17
**Chromosome II**	Arsenical resistance operon repressor	1	ABC transporter, ATP-binding protein	1
	Autotransporter adhesion	1	Acetyltransferase GNAT family	1
	Histone acetyltransferase HPA2	1	Branched-chain amino acid aminotransferase	1
	Cupin CDS	1	Death on curing protein, Doc toxin	1
	DNA damage-inducible protein DinB	1	Error-prone repair protein UmuC	1
	DUF1706 domain-containing protein	1	Flavohemoprotein (Hemoglobin-like protein)	1
	Glyoxalase family protein	2	GCN5-related N-acetyltransferase	1
	Inner membrane protein YrbG	1	Glycogen-debranching protein	1
	Iron aquisition yersiniabactin synthesis enzyme (Irp3)	1	Mobile element protein	1
	Iron aquisition yersiniabactin synthesis enzyme (YbtT)	3	N-acetylneuraminic acid mutarotase	1
	L-fucose mutarotase	1	NADH:flavin oxidoreductase	1
	Methionine ABC transporter ATP-binding protein	1	PbpG	1
	N-acetylglucosamine-6-phosphate deacetylase	1	Phenazine biosynthesis protein PhzF	1
	Oxidoreductase	2	Thiosulfate sulfurtransferase	1
	Putative integral membrane protein	1	Hypothetical protein	32
	Putative tautomerase	1		
	Sialic acid-induced transmembrane protein YjhT	1		
	Transcriptional regulator PaiB-like	1		
	Transcriptional regulator TetR family	1		
	Unknown gene	2		
	Hypothetical protein	56		

The numeric value represents the additional number of the genes detected in each strain.

## Discussion

In recent years, molecular techniques have been used for the study of bacteria and to establish phylogenetic relationships among them. This study has employed various molecular methodologies to determine the differences between three strains of *V*. *mimicus* isolated from the washing step in a shrimp processing facility in Sonora, Mexico. The *vmh* gene is common to *V*. *mimicus* and is a useful marker for species identification [27,28]. Different studies have used some of these methods, but it has not been reported before, the simultaneous use of all these techniques for the purpose to study single species and establish which method is more useful to detected genetic variations [37–40]. In this study, ERIC-PCR and RAPD (*vmh*390R) were the most discriminatory techniques for establishing differences, by obtaining the lowest percentages of similarities between the analyzed strains, while GTG_5_-PCR was the less discriminatory method. ERIC-PCR has been reported for the study of several bacteria, such as *V*. *cholerae*, where different patterns were detected, with up to 8 amplification products [38,41]. This is the first report where ERIC-PCR was used for the analysis of *V*. *mimicus* strains and by obtaining up to 13 amplified products; this method proved to be a powerful tool for the study of this species and can be used for the detection of genetic variability. Bi *et al*. [37] used the arbitrarily primed polymerase chain reaction (AP-PCR) to study several clinical strains of *V*. *mimicus*, and when comparing their results with those of this study, a similar number of band patterns were found, with the exception that their amplified products were of larger size. It is worth mentioning that the strains used in the previous study were clinical, while in this study, the strains were environmental. Additionally, it is know that there are differences in the genome of clinical and environmental strains of this species [16], so it is possible that these differences can explain the differences in the number of the amplified products. Urakawa *et al*. [39] performed an RFLP analysis with the 16S rRNA genes of several species of *Vibrio*, including *V*. *mimicus* type strain (CAIM 602^T^). Their study used several restriction enzymes, including *Hae*-III, *Hin*-fI and *Alu*-I, but the results using these enzymes were omitted because of ambiguous results. In this study, these same enzymes also showed a low level of differentiation between strains, compared with the enzymes *Cfo*-I and *Hap*-II, which showed a better differentiation between the strains isolated from the frozen shrimp processing facility. This is the first report in which the enzymes *Cfo*-I and *Hap*-II were used for the study of strains of *V*. *mimicus*. However, it was not possible to differentiate between *V*. *mimicus* CAIM 602^T^ and *V*. *mimicus* 93 with these enzymes. In addition, with the cluster analysis that evaluated all the methodologies (ERIC-PCR, RAPD, GTG-_5_ and ARDRA); it was possible to confirm that the three analyzed strains were different even when they were isolated from the same source. In addition, it is important to consider that each method provides different patterns-resolution, and the selection of one should depend in the desired resolution.

Genome sequencing is opening a door for the understanding of bacteria pathogenesis and study evolutionary history [42]. This study found differences in the gene content in both environmental strains of *V*. *mimicus* (CAIM 1882 and CAIM 1883). Those differences were mostly in the ChII involving proteins and virulence genes. Furthermore, the proteome matrix point out differences between these genomes, even when the strains were obtained from the same niche at the same time. These findings give us an outlook of the genomic diversity of *V*. *mimicus*.

These environmental genomes presented several virulence genes that were classified into 16 categories, with type I secretion system proteins as the main category. Additionally, genes for type IV pilus proteins (MSHA pilus) and extracellular components such as the capsular polysaccharide (CPS) were present, which have been reported in few studies of *V*. *mimicus* [13,16]. Furthermore, this is the first report of an accessory colonization factor (*acf*D gene) found in *V*. *mimicus*, that is also present in others *Vibrio* species, such as *V*. *cholerae*, *V*. *fischeri*, *V*. *vulnificus* and *V*. *parahaemolyticus*, which have been documented to be required for an efficient intestinal colonization, and disruption of any of the four *acf* genes (*acf*A, B, C, D) can reduce the ability to colonize [43–48]. Sultan *et al*. [49] found the presence of AI-2 activity and the *lux*S, *lux*O and *lux*R genes in clinical strains of *V*. *mimicus*, and in this study the same genes were found in environmental strains of *V*. *mimicus*. However, no assays were performed so it remains to be determined their exact roles in which they function. Also, various genes such as the aerobactin siderophore, the ferric aerobactin receptor, ToxR, ToxS, and several others genes found in these strains, have been previously reported in others strains of *V*. *mimicus* [27,50,51]. Recently, it was reported the adhesion function of the *Omp*U protein of *V*. *mimicus* [52]. Moreover, various virulence genes found in this study (e.g. *Omp*U, *acf*D, MSHA pilus, δ-VPH*;*, CPS, ToxS, ToxR, etc.) have been studied in other *Vibrio* species, where their pathogenic mechanisms had been reported, but in *V*. *mimicus* little is known about it and more studies are required for establishing the mechanisms that explain the virulence of *V*. *mimicus* [53–57]. Additionally, a phylogenetic analysis of these virulence genes established a relationship between these environmental genomes. In spite of the lack of virulence mechanisms studies and because the close relationship between *Vibrio* species, we could expect that those genes will have similar virulence mechanisms in *V*. *mimicus*. Therefore the presence of this bacterium in environment could represent a risk to the health of the shrimp consumers, especially because this food can be consumed raw. In addition, the identification of several virulence genes in these strains, that are also present in other *Vibrio* species, support previous studies that suggest that *V*. *mimicus* could be a gene reservoir in the environment for *Vibrio* species [19,20].

In conclusion, the information described in this study substantiated the importance and effectiveness of the molecular and genomic methodologies to study and characterize microorganisms. Differences between the analyzed environmental strains were found, even though they came from the same source, offering a perspective of the genomic diversity of *V*. *mimicus*. Several virulence genes were identified, supporting the pathogenic potential of this species and a phylogenetic relationship between some core genome virulence genes were established. However, more studies are required to determine how these detected virulence genes may have or not an effect on the health of shrimp consumers. Further studies of genes of clinical importance will help to better understand their potential virulence, environmental impact and evolution.
